# Modulation of vascular contraction via soluble guanylate cyclase signaling in a novel ex vivo method using rat precision‐cut liver slices

**DOI:** 10.1002/prp2.768

**Published:** 2021-05-20

**Authors:** Anouk Oldenburger, Gerald Birk, Marco Schlepütz, Andre Broermann, Birgit Stierstorfer, Steven S. Pullen, Jörg F. Rippmann

**Affiliations:** ^1^ CardioMetabolic Diseases Research Boehringer Ingelheim Pharma GmbH & Co. KG Biberach a.d. Riss Germany; ^2^ Target Discovery Sciences Boehringer Ingelheim Pharma GmbH & Co. KG Biberach an der Riss Germany; ^3^ Immunology and Respiratory Diseases Research Boehringer Ingelheim Pharma GmbH & Co. KG Biberach an der Riss Germany; ^4^ CardioMetabolic Diseases Research Boehringer Ingelheim Pharmaceuticals, Inc Ridgefield CT USA; ^5^ Cancer Immunology+Immune Modulation Boehringer Ingelheim Pharma GmbH & Co. KG Biberach a.d. Riss Germany

**Keywords:** blood vessel contraction, cGMP, ex vivo model, NASH, portal hypertension, translation

## Abstract

Fibrotic processes in the liver of non‐alcoholic steatohepatitis (NASH) patients cause microcirculatory dysfunction in the organ which increases blood vessel resistance and causes portal hypertension. Assessing blood vessel function in the liver is challenging, necessitating the development of novel methods in normal and fibrotic tissue that allow for drug screening and translation toward pre‐clinical settings. Cultures of precision cut liver slices (PCLS) from normal and fibrotic rat livers were used for blood vessel function analysis. Live recording of vessel diameter was used to assess the response to endothelin‐1, serotonin and soluble guanylate cyclase (sGC) activation. A cascade of contraction and relaxation events in response to serotonin, endothelin‐1, Ketanserin and sGC activity could be established using vessel diameter analysis of rat PCLS. Both the sGC activator BI 703704 and the sGC stimulator Riociguat prevented serotonin‐induced contraction in PCLS from naive rats. By contrast, PCLS cultures from the rat CCl_4_ NASH model were only responsive to the sGC activator, thus establishing that the sGC enzyme is rendered non‐responsive to nitric oxide under oxidative stress found in fibrotic livers. The role of the sGC pathway for vessel relaxation of fibrotic liver tissue was identified in our model. The obtained data shows that the inhibitory capacities on vessel contraction of sGC compounds can be translated to published preclinical data. Altogether, this novel ex vivo PCLS method allows for the differentiation of drug candidates and the translation of therapeutic approaches towards the clinical use.

## Graphical abstract

Cultures of rat precision cut liver slices (PCLS) were used for ex vivo blood vessel function analysis via live recording. The sGC activator BI703704 differentiates from the sGC stimulator Riociguat in the prevention of serotonin induced blood vessel contraction of normal and fibrotic rat liver slices.

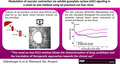

**Abbreviations**
3R: reduce, refine, replace
BDL: bile duct ligation
CCl_4_: carbon tetrachloride
cGMP: cyclic guanosine monophosphate
eNOS: endothelial Nitric Oxide Synthase
HMOX1: haem oxygenase 1
HVPG: hepatic venous pressure gradient
NAFLD: non‐alcoholic fatty liver disease
NASH: non‐alcoholic steatohepatitis
NFE2L2: nuclear factor, erythroid 2 like 2
NO: nitric oxide
NQO1: NAD(P)H Quinone Dehydrogenase 1
PCLS: precision‐cut liver slices
sGC: soluble Guanylate Cyclase
UW: University of Wisconsin
VASP: vasodilator‐stimulated phosphoprotein

**Summary**

**What is already know?**

- Precision cut liver slices conserve hepatic physiology and response to fibrotic stimuli
- Limiting methods to analyze blood vessel function available, raising the need for new translatable methods
- Portal hypertension as a response to fibrotic processes in NASH patients.

​

**What did this study adds?**

- A description of a pharmacological treatment and analysis of hepatic vessel function under fibrotic conditions.
- Demonstration of the sGC activator induced vasodilation under oxidative stress conditions in the liver.

​

**Clinical significance**

- The ex vivo method allows for pre‐clinical to clinical translation of pharmacodynamic effects.

​

​

## 1 INTRODUCTION

Nonalcoholic fatty liver disease (NAFLD) encompasses a disease spectrum from excessive hepatic fat accumulation to chronic liver inflammation (nonalcoholic steatohepatitis (NASH)) and around 24% of the population in Europa has NAFLD.^1^

Accumulation of triglycerides in hepatocytes as seen in NAFLD^2^ leads to initiation of inflammation and fibrosis. Reactive oxygen species (ROS) as part of the inflammatory response induce vascular remodeling, fibrosis, and wound healing processes^3^ which drive NALFD progression to NASH.^3^ Fibrosis can eventually lead to liver cirrhosis, a condition where there is loss of cell organization leading to reduced liver function,^3^ microcirculatory dysfunction,^4^ and alterations in the blood vessel architecture. These structural changes generate elevated pressure in the portal vein^5^ in which elevated levels of vasoconstrictors such as endothelin‐1 and serotonin contribute.^6^, ^7^ Currently, there is no treatment for portal hypertension.^8^ Evaluation and differentiation of new drug candidates for the treatment of portal hypertension require a translatable model.

A widely used and direct in vivo measurement of portal pressure is achieved by catheterization of the portal vein^9^ and analysis of the hepatic venous pressure gradient (HVPG).^9^ However, this direct measurement is invasive, difficult, and allows only a low throughput. In the present study, we established an ex vivo method based on precision‐cut liver slices (PCLS) to directly assess blood vessel dilation and contraction in liver explants.

Contraction has been induced in our ex vivo method by two potent modulators of liver vascular tone, serotonin (5HT) and endothelin‐1 (ET‐1).^10^, ^11^ ET‐1 is increased in NASH patients, serum levels correlate with the severity of fibrosis, and blocking ET‐1 decreases fibrosis making it of interest when investigating therapeutic concepts for NASH.^12^ The beneficial effect of targeting the serotonin pathway is already established with the use of a 5‐HT_3_ receptor antagonist which could ameliorate steatosis and inflammation in the liver of *ob*/*ob* mice.^13^

A potential therapeutic target to enhance vasodilation and thereby reduce portal hypertension is soluble guanylate cyclase (sGC), an enzyme acting as a receptor for the vasodilator nitric oxide (NO). Binding of NO to sGC leads to the generation of cyclic guanosine monophosphate (cGMP), an important second messenger that induces vascular smooth muscle cell relaxation and blood vessel dilation.^14^, ^15^

The functional sGC enzyme consists of an α‐subunit (α1 or α2) and a β‐subunit (β1 or β2) containing a haem prosthetic group which is responsible for NO‐sensing when the iron is in a reduced state. A conformation change induced by NO binding to the ferrous iron results in stimulation of catalytic activity while oxidation of the haem under conditions of oxidative stress renders sGC insensitive to NO.

Increased enzymatic activity of the sGC enzyme can be achieved by either sGC stimulators which increase the sensitivity of the haem prosthetic group for NO or sGC activators that function independently of NO on haem‐oxidized or haem‐free sGC enzyme.^16^ Oxidation of the haem under conditions of oxidative stress as observed in NASH patients has the potential to render the sGC enzyme insensitive to NO^17^ making an sGC activator potentially more effective than an sGC stimulator. For the comparison of sGC stimulators and activators, we used published representative compounds. Riociquat, an sGC stimulator, has shown efficacy as a vasodilator in a phase III trial for pulmonary arterial hypertension and chronic thromboembolic pulmonary hypertension^18^ and as a treatment for portal hypertension. The compound BI 703704, known to modulate sGC by dose‐dependently increasing cGMP levels independent of the oxidative status of the enzyme, showed in vivo potency in the treatment of diabetic nephropathy and portal hypertension.^19^, ^20^

We sought to explore the translation of the beneficial role of sGC in treating portal hypertension in vivo and its link to serotonin‐induced blood vessel contraction in a new ex vivo model. PCLS have been efficiently used for the characterization of fibrotic pathways and the effect of compound treatments.^21^, ^22^ Here we established their use for the investigation of compound‐related effects on serotonin‐induced vessel contraction using Ketanserin and sGC‐related compounds. Both an sGC activator and an sGC stimulator were able to induce a dose‐dependent inhibition of blood vessel contraction using PCLS from normal mice. Using fibrotic liver tissue, the sGC activator showed a superior effect in reducing contraction under diseased conditions with elevated oxidative stress.

Overall, the PCLS model system allows for the analysis of vessel diameter and the prioritization of potential therapeutics for portal hypertension and the translation to more complex in vivo models.^20^

## 2 MATERIALS AND METHODS

### 2.1 Materials

Coring press was obtained from Alabama Research & Development. University of Wisconsin (UW) organ preservation solution was from DuPont Critical Care, Waukegab, IL, USA. Krebs‐Henseleit buffer modified (K3753), NaHCO_3_, d‐Glucose (45%), HEPES, 1× HBSS, EDTA Ketanserin (+)‐tartrate salt # S006‐50MG and Endothelin‐1 (ET‐1) # E7764‐1 mg all obtained from Sigma Aldrich. Williams E medium was from Lonza. Glutamax and gentamicin were provided by Gibco. FastPrep System tubes were obtained from MP Biomedicals. ATP Bioluminescence Assay Kit was provided by Roche. Pierce™ protein assay kit was obtained from Thermo Scientific.

RLT buffer was from Qiagen. High capacity cDNA Archive Kit was obtained from Applied Biosystems (#4322169). The quantitative real‐time PCR with gene‐specific primers was from Applied Biosystems. The following primers were used *Nfe2 l2* (Rn00582415_m1), *Nqo1* (Rn00566528_m1), *Hmox1* (Rn00561387_m1) and *rRNA Pol2* (Rn01752026_m19).

### 2.2 Animals

Male Wistar rats (300–400 g) from Charles River Research Models and Services Germany GmbH were used for all control experiments. Male rats for selected for this study based on the know differences observed between the severity of liver damage induced by CCl4 in male and female rats.^23^ In Figures 2 and 3, healthy Wistar rats are used to measure the contraction of blood vessels in PCLS. Sprague‐Dawley rats are used for the CCl4 in vivo model.

Animals were housed pairwise in a controlled environment (21°C ± 2°C, humidity 50% ± 10%) with a 12‐h light/dark cycle and fed KLIBA NAFAG 3438 (chow) ad libitum.

Rats were euthanized without fasting by pentobarbital anesthesia and exsanguination at the beginning of the light cycle. The liver was removed and kept on ice in the University of Wisconsin (UW) organ preservation solution (DuPont Critical Care) until PCLS preparation. The animal studies were conducted according to approved animal licenses of Boehringer‐Ingelheim and conform to national guidelines for animal welfare.

### 2.3 CCl_4_ model

Male Sprague‐Dawley rats purchased from Janvier Labs, CS 4105‐Le Genest‐Saint‐Isle, 53941 Saint‐Berthevin Cedex, France were treated with carbon tetrachloride (CCl_4_). Male rats were selected for this study based on the severity of the male rat CCl4 models which represent features of NASH patients. Stock solution 100% CCl_4_ was diluted 1:10 in olive oil and applied at 0.25 ml/kg p.o. The application was three times a week for 8 weeks to induce fibrosis. An olive oil control group was included in the experiment. In Figures 4, 5, 6, 7, Sprague‐Dawley rats are used, either vehicle or CCl4 treated. The difference between the use of two different strains of rats is due to the chosen NASH in vivo model. The CCl4 model is established in Sprague‐Dawley rats and the vehicle control group was derived from this same strain.

### 2.4 Compounds

5‐HT and endothelin‐1 (ET‐1) were used to induce a contraction response in PCLS. As seen in Figure 2 an EC50 of 1 µM 5‐HT and of 1 mM ET‐1was calculated. In order to induce rapid, full, and long‐term response 1 mM 5‐HT and 25 mM ET‐1 was used in the follow‐up studies for the induction of contraction. The relaxation of the vessels was tested with the preincubation of Ketanserin, a 5‐HT receptor antagonist, Riociquat and BI00703703. All three compounds were used at a concentration of 10 µM.

**FIGURE 1:**
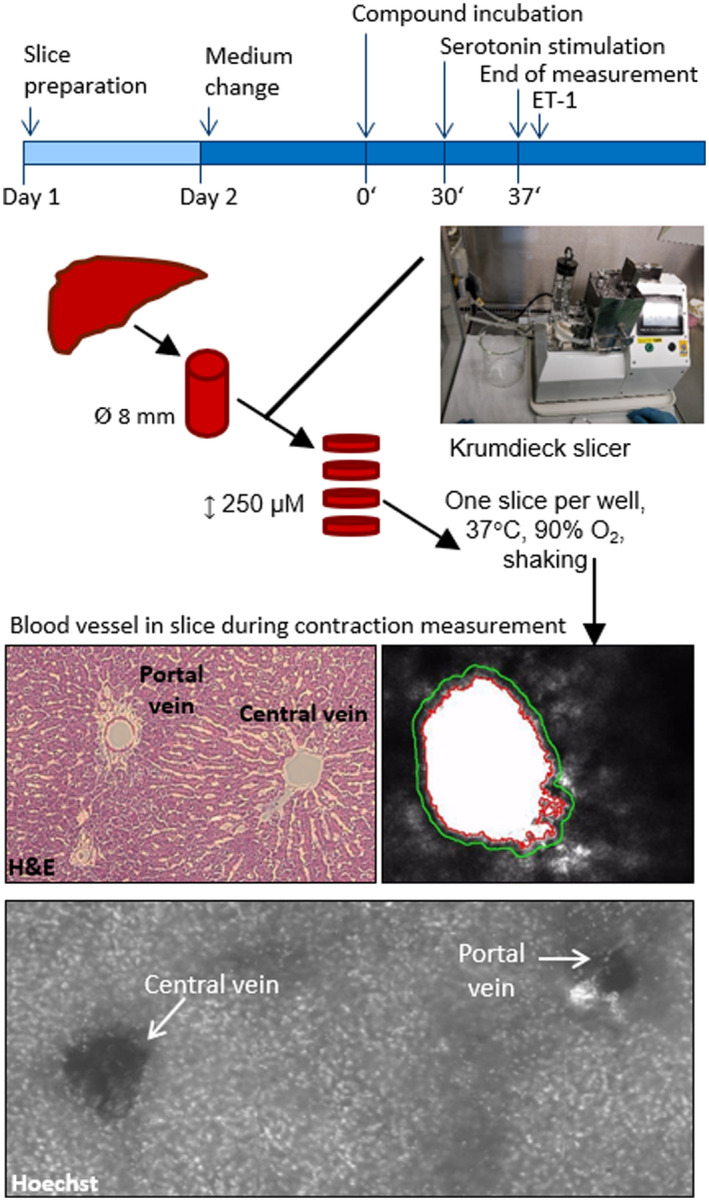
Ex vivo model for measuring blood vessel contraction in precision‐cut liver slices (PCLS). Tissue cores were punched out from rat livers. PCLS were prepared by means of a Krumdieck tissue slicer. Each slice was incubated at 37°C under high oxygen and shaking conditions. The day after slicing medium was changed. Slices containing blood vessels were selected and incubated with 1 µM or 10 µM compound for 30 min at 37°C. PCLS were transferred into cavities of standard multi‐well cell culture plates, mounted by a platinum ring to prevent floating and subjected to video‐microscopy. Serotonin was added to PCLS and pictures were taken every 10 s for 7 min. Blood vessel area was analyzed by Axiovision Software. H&E and Hoechst staining of PCLS showed the presence of the portal vein and the central vein in the slices

Ketanserin had an EC50 of 9 nM in K+‐induced contractions of the rat aortic strip^24^ and an EC50 of 0.5 µM in 5‐HT induced contractions of the rat aortic strip.^25^

A concentration–response curve of Riociguat showing a dose of 10 µM inducing a near‐maximal relaxation in rat pulmonary artery rings. An EC_50_ of 130 nM was shown.^26^ BI 703704 showed an EC_50_ of 105 nM in renal cGMP content measured in diabetic rats.^19^ Using a 10 µM concentration will show a maximal effect.

### 2.5 Preparation of PCLS

Tissue cores with a diameter of 8 mm were punched from rat livers using a coring press (Alabama Research & Development) and stored in University of Wisconsin (UW) solution on ice. Slices with a thickness of approximately 250 µm were prepared with a pre‐cooled Krumdieck slicer (Alabama Research & Development) in ice‐cold Krebs–Henseleit buffer (Krebs–Henseleit buffer modified (K3753) supplemented with 25 mM NaHCO_3_, 25 mM d‐glucose (45%), 10 mM HEPES (all obtained from Sigma Aldrich) (Figure 1). Each slice was incubated at 37°C under 90% oxygen and shaking conditions in a 12 well plate containing 1.3 ml of Williams E medium (Lonza) with additives (2 mM Glutamax (Gibco), 25 mM D‐Glucose (45%) and 50 µg/ml gentamicin (Gibco)) per well (Figure 1).

### 2.6 Blood vessel contraction and relaxation: set‐up and analysis

Slices containing blood vessels were selected and pre‐incubated with DMSO, Ketanserin (+)‐tartrate salt (Sigma Aldrich, # S006‐50MG), Riociguat or BI 703704 at 90% O_2_, shaking conditions and 37°C for 30 min before they were placed under the microscope (Zeiss, Axiovert 135TV). Slices were immobilized with a platinum ring before the start of the experiment. 1 mM serotonin (Sigma Aldrich, # 14927–25 mg) was added to the slices to induce contraction and pictures of the blood vessel were taken every 10 s for 7 min using Axiovison software. Endothelin‐1 (ET‐1) (Sigma Aldrich, # E7764‐1 mg) at a final concentration of 10 mM was added to the slice as a positive control for the contractibility of the blood vessel. Only blood vessels that responded to ET‐1 treatment were included in the final analysis. Blood vessel area was analyzed using a proprietary application based on a commercially available machine vision software library (Halcon 13.0.2, MVTec Software GmbH). The area of the unstimulated vessel was set as 100% and used for normalization.

### 2.7 Histology

After the contraction experiment, slices were fixed in 4% paraformaldehyde or directly stained. Slices were placed in incubation buffer (1× HBSS, 8.9 mM sodium bicarbonate, 5% fetal bovine serum, Sigma Aldrich) containing 5 µg/ml Hoechst for 30 min shaking at 4°C followed by a washing step of 20 min in fresh incubation buffer. For analysis, slices were placed on an object slide and covered using a mounting medium. Hematoxylin and eosin (H&E) staining was performed on liver slices (3 µm thickness) embedded in paraffin. Slides were scanned with a Zeiss Axio Scan.Z1.

### 2.8 Viability assay: ATP

Directly after slicing and after 24 h of incubation, PCLS were collected for ATP measurements in Precellys^®^ Keramik‐Kit 1 mm tubes in 1 ml sonification buffer containing 70% ethanol and 2 mM EDTA (Sigma Aldrich). Slices at 0 h were collected at 4°C, which is the temperature during slicing, but slices at 24 h are collected at 37°C. Homogenization of PCLS was achieved with the use of the FastPrep System from MP Biomedicals and followed by centrifugation (18000*g*, 5 min, 4°C). ATP levels were measured by ATP Bioluminescence Assay Kit (Roche) in the supernatants while the pellets were used for protein determination. The pellets were dried and homogenized in 1 M NaOH. Protein amounts were determined by Pierce™ protein assay kit (Thermo Scientific).

### 2.9 RT‐PCR

PCLS were stored at −20°C in Precellys^®^ Keramik‐Kit 1.4 mm tubes containing 400 µl RLT (Qiagen) +1% β‐mercaptoethanol. Slices were lysed using the Precellys Evolution twice at 2600 g for 30 s each. 400 µl of 70% ethanol was added before mRNA isolation according to the RNAeasy protocol from Qiagen. Following the manufacturer protocol, 1 µg RNA was transcribed into cDNA using High capacity cDNA Archive Kit from Applied Biosystems (#4322169). The cDNA was amplified in a quantitative real‐time PCR with gene‐specific primers from Applied Biosystems. The following primers were used *Nfe2l2* (Rn00582415_m1), *Nqo1* (Rn00566528_m1), *Hmox1* (Rn00561387_m1), *Gucy1A3* (Rn00567252_m1), *Gucy1a2* (Rn00675050_m1), *Gucy1b3* (Rn00562775_m1), *Gucy1b2* (Rn01636981_m1) and *rRNA Pol2* (Rn01752026_m19). SDS software (Version 2.2, Applied Biosystems) was used for the evaluation of the results. Relative quantities of expression levels were determined by comparison of ct‐values of the samples with ct‐values of a dilution series of a standard total RNA sample. For the normalization, relative quantities of a transcript were divided by the relative quantities of the corresponding RNA Polymerase II transcript.

### 2.10 Data and statistical analysis

The data and statistical analysis comply with the recommendations on experimental design and analysis in pharmacology.^27^

Group size was based on a predictive effect size of 15% contraction in our control group with a mean of 100% and SD of 8%. A group size of *n* > 5 is needed with α: 0.05 and β: 0.80.

At the end of each contraction experiment, the contractile response of each slice was determined by the addition of ET‐1. Due to this stringent quality control, which was applied after the samples were taken, several samples did not reach the final analysis. In accordance with this high internal standardization and quality control for the vessel function, the experiments allow for the reduced number of samples.

Another deviation concerning group size is seen in the DMSO control group in the contraction experiment, where more than n:5 were included. As different rats were used, we put a high value to test multiple DMSO controls for each rat to obtain a solid characterization of each rat using the new setup before testing compound incubation on the other slices for each rat. DMSO control was taken at different times of the day to overcome the variation timing possibly can cause.

Data are presented as mean ± SEM. Two groups were compared by unpaired *t*‐test, whereas more than two groups were analyzed by one‐way ANOVA and corrected for multiple comparisons by Dunnett's test. The pairing was effective in repeated measurements. *p*‐values <.05 were considered significant and indicated with * or § depending on the comparison. Figure legends refer to the specific test used. Concentration‐response curves were calculated by 4‐parameter logistic regression. Time‐courses were transformed to a univariate analysis by calculating the area‐under‐the‐curve (AUC). GraphPad Prism 8 software was used for analysis.

Contraction experiments were normalized to the diameter of each blood vessel at the beginning of the experiment. This diameter was set on 100% and contraction during the experiment was in comparison to the normalized baseline diameter of 100%. Due to variation in the diameter between the different blood vessels normalization was required where the diameter of each blood vessel at the start of the experiment was set to 100%.

## 3 RESULTS

### 3.1 Serotonin‐induced contraction in blood vessels of PCLS is prevented by a serotonin 5HT_2_ receptor antagonist

Ex vivo cultures of defined liver slices have been used already for the characterization of fibrotic processes and intervention by pharmacological treatment.^21^, ^22^ In extension to this application, we established the analytics and assay conditions that allowed the study of real‐time contraction and relaxation processes of liver blood vessels.

To identify the presence of the portal and central vein in PCLS, H&E staining was used. A clear distinction could be made as the portal vein has a bile duct and artery in proximity (Figure 1). A similar observation was made when using Hoechst staining to visualize the nucleus of cells (small bright dots in the picture of Figure 1). A more pronounced bright staining was observed near the portal vein as an indicator for the presence of a bile duct and an artery. Although identification of the different veins is possible with histology, the difference between the central vein and portal vein cannot be seen with live imaging during contraction experiments. Therefore, both central and portal veins are included in the analysis.

**FIGURE 2:**
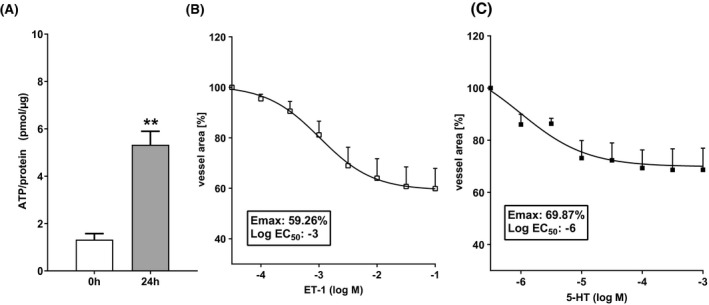
Serotonin contract vasculature in PCLS. (A) ATP levels measured directly after PCLS preparation (0 h) and after 24 h of incubation at 37°C under high oxygen conditions (n:3). (B) Concentration–response curve of endothelin‐1‐induced blood vessel contraction in PCLS after 24 h of incubation (n:8). (C) Concentration–response curve of serotonin‐induced blood vessel contraction in PCLS after 24 h of incubation (n:5). Data are presented as mean ± SEM. **p < .01 compared to 0 h group (unpaired *t*‐test)

In the first step, the viability of the slices was assessed and confirmed by ATP measurements (Figure 2A). After 24 h, an approximately threefold increase in ATP levels was observed compared to the levels present in PCLS immediately after slicing. Thereafter, the response to classical contraction and relaxation stimuli was established. After the slicing of the tissue, PCLS were conditioned with a standard medium for 24 h prior to the induction of contraction by either ET‐1 or serotonin. Both ET1 and serotonin contraction of vessels in PCLS was concentration‐dependent (Figure 2B and C) and the contraction reached a steady‐state initial blood vessel area after 7 min.

**FIGURE 3:**
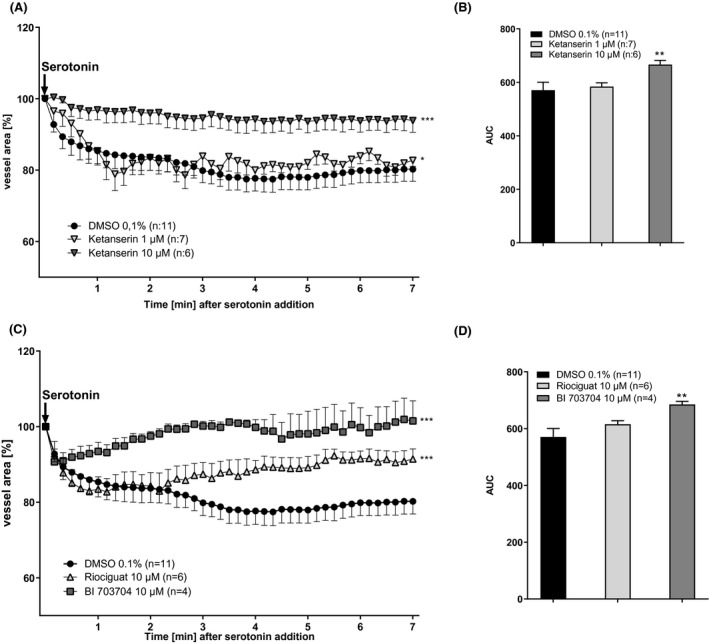
Ketanserin and sGC compounds prevent serotonin‐induced blood vessel contraction in PCLS. (A) PCLS were pre‐incubated with 1 µM or 10 µM Ketanserin for 30 min followed by stimulation with 1 mM serotonin for 7 min. Every 10 s a picture was taken and blood vessel area was measured. (B) Analysis of area under the curve. (DMSO n:11, Ketanserin 1 µM n:7, Ketanserin 10 µM n:6). (C) PCLS were pre‐incubated with 1 µM or 10 µM Riociguat or BI 703704 for 30 min followed by stimulation with 1 mM serotonin for 7 min. Every 10 s a picture was taken and blood vessel area was measured. (D) Analysis of area under the curve. (DMSO n:11, Riociguat 10 µM n:6, BI 703704 10 µM n:4). Technical loss of measurements has occurred. **p < .01 compared to DMSO 0.1% (One Way ANOVA for (A) and (C), Mann–Whitney for B and D)

To examine if the serotonin response can be prevented, PCLS were pre‐incubated for 30 min with either 0.1% DMSO (solvent control) or different concentrations of Ketanserin, a 5‐HT_2_ receptor antagonist,^28^ followed by stimulation with 1 mM serotonin. Pre‐incubation of the slices with 10 µM Ketanserin, completely blocked the serotonin‐induced contraction in comparison to pre‐incubation of the slices with 0.1% DMSO (Figure 3A). Analyzing the area under the curve (AUC) for the three different incubation conditions, (Figure 3B) showed a concentration‐dependent inhibition of serotonin‐induced contraction by Ketanserin.

### 3.2 Both the sGC stimulator Riociguat and the activator BI 703704 prevent serotonin‐induced contractions in blood vessels in healthy PCLS

To investigate the role of the sGC pathway in the contraction of blood vessels, PCLS were pre‐incubated with 10 µM Riociguat (sGC stimulator) or 10 µM BI 703704 (sGC activator).^19^

The contraction induced by serotonin was completely blocked by BI 703704 (Figure 3C and D) early after the addition of serotonin. In contrast, Riociguat was not able to fully inhibit vessel contraction in PCLS and there was a delayed response compared to BI 703704 treatment (Figure 3C and D).

### 3.3 PCLS from CCl_4_‐treated animals show changes in sGC subunit expression, oxidative stress response, and fibrosis signatures

To determine if the difference between the sGC stimulator and sGC activator is more evident under oxidative stress conditions, PCLS from rats treated with CCl_4_ were prepared. Sprague Dawley rats were used for our CCl_4_ model as these rats are more prone to a cirrhotic phenotype compared to Wistar rats.^29^

**FIGURE 4:**
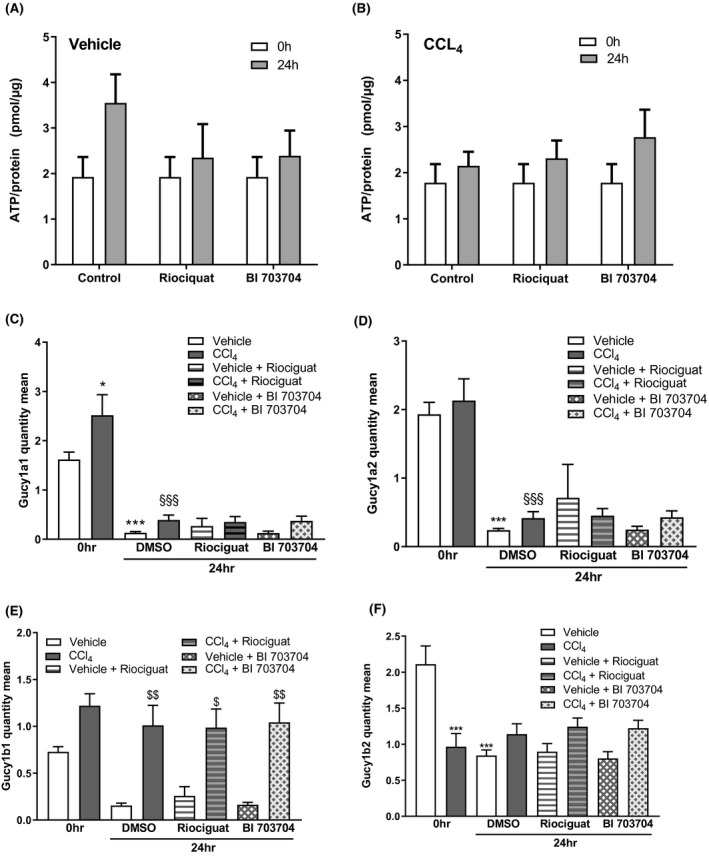
Expression of sGC subunits in PCLS from the vehicle and CCl_4_‐treated animals. (A) ATP levels were measured directly after PCLS preparation (0 h) and after 24 h incubation at 37°C under high oxygen conditions. ATP results from vehicle‐treated animals (A) and CCl_4_‐treated animals (B). mRNA expression of α1 subunit of the sGC enzyme (C), α2 subunit (D), β1 subunit (E) and β2 subunit (F) in PCLS stimulated with sGC compounds for 24 h in vehicle‐ and CCl_4_‐treated animals. n: 5 for all groups. *p < .05, **p < .01, ***p < .001 compared 0 h DMSO group. ^§^ *p* < .05, ^§§^ *p* < .01, ^§§§^ *p* < .001 compared to DMSO at 24 h (One‐way ANOVA, Dunnett's post test)

The expression of all four sGC subunits was analyzed in PCLS from vehicle and CCl_4_‐treated animals (Figure 4). In PCLS from the CCl_4_ model, an increase in the expression of α1 subunit (Figure 4C), and a trend towards an increase in β1 subunit expression (Figure 4E) was observed. The α2 subunit (Figure 4) was not altered by CCl_4_ treatment while the β2 subunit (Figure 4F) was decreased in PCLS of CCl_4_ animals. After 24 h of incubation of PCLS, a decrease in expression of the α1 subunit (Figure 4) and the α2 subunit (Figure 4D) was seen for both PCLS from vehicle and CCl_4_‐treated animals. The β1 subunit (Figure 4E) was only decreased after 24 h in the vehicle group but not in the CCl_4_ group. The vehicle group also showed a decrease of the β2 subunit (Figure 4F) after 24 h. This subunit is already decreased after CCl_4_ treatment but no further decrease was observed after incubation. Incubation of the slices with either Riociguat or BI 703704 did not alter the expression of the four subunits (Figure 4C–F).

**FIGURE 5:**
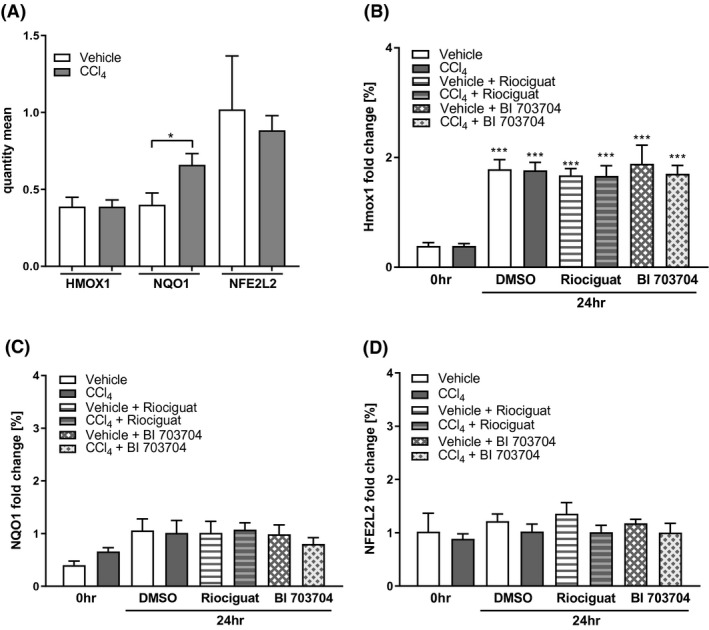
Expression of oxidative stress markers is increased in PCLS from CCl_4_‐treated animals. (A) Basal expression of Hmox1, NQO1, and Nfe2L2 in PCLS from vehicle‐ and CCl_4_ ‐treated animals. mRNA expression of Hmox1 (B), NQO1 (C) and Nfe2L2 (D) in PCLS from vehicle‐ and CCl_4_‐treated rats after 24 h of incubation compared to the quantity measured at 0 h levels. Data presented as mean ± SEM. n: 5 for all groups. *p < .05, **p < .01, ***p < .001 compared 0 h DMSO group. ^§^ *p* < .05, ^§§^ *p* < .01, ^§§§^ *p* < .001 compared

CCl_4_ was used as a model for NASH in which oxidative stress is increased. Reactive‐oxygen species (ROS) are neutralized by protective mechanisms^30^ which are induced by the transcription factor Nrf2: These include haem oxygenase‐1 (HMOX‐1) and NAD(P)H:quinone dehydrogenase 1 (NQO1).^30^ We investigated the mRNA expression of certain oxidative stress markers in PCLS (Figure 5). Figure 5A shows that NQO1 is increased in PCLS from CCl_4_‐treated rats compared to vehicle‐treated rats at 0 h. After incubation for 24 h HMOX1 was increased in PCLS from vehicle and CCl_4_‐treated animals (Figure 5B). NQO1 was already increased in CCl_4_‐treared rats and is not further increased upon 24 h incubation. No changes were observed in NFE2L2 after CCl_4_ treatment or due to incubation of PCLS (Figure 5).

Furthermore, slices from CCl_4_‐treated animals showed an increase in the expression of fibrotic mRNA markers such as collagen 1α1 and α‐smooth muscle actin (Figure S1). The viability of the slices in both the vehicle and CCl_4_‐treated animals was not significantly changed after 24 h of incubation (Figure 4A and B).

### 3.4 Blood vessels in PCLS obtained from CCl_4_‐treated animals showed a reduced contractile response to serotonin

**FIGURE 6:**
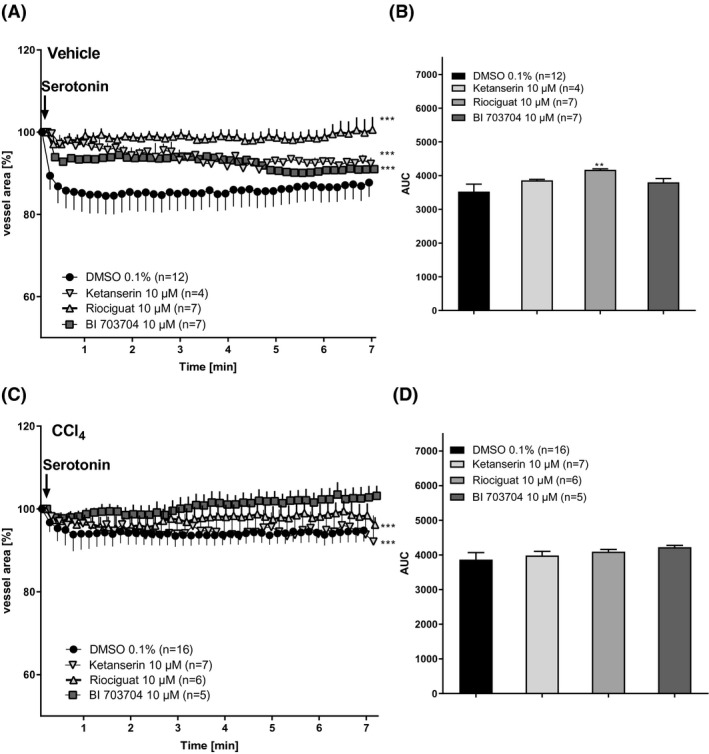
PCLS from CCl_4_‐treated rats show reduced serotonin‐induced contraction compared to PCLS from naïve rats. (A) PCLS of vehicle‐treated animals were pre‐incubated with 10 µM of Ketanserin, Riociguat, or BI 703704 for 30 min followed by stimulation with 1 mM serotonin for 7 min. Every 10 s a picture was taken and blood vessel area was measured. (B) Area under curve in vehicle‐treated animals. (DMSO n:12, Ketanserin 10 µM n:4, Riociguat 10 µM n:5, BI 7030704 10 µM n:7). Technical loss of measurements has occurred. (C) PCLS of CCl_4_‐treated animals were pre‐incubated with 10 µM Ketanserin, Riociguat, or BI 703704 for 30 min followed by stimulation with 1 mM serotonin for 7 min. Every 10 s a picture was taken and blood vessel area was measured. (D) Area under the curve in PCLS from CCl_4_‐treated animals. (DMSO n:17, Ketanserin 10 µM n:7, Riociguat 10 µM n:6, BI 7030704 10 µM n:5). *p < .05, **p < .01, ***p < .001 compared to DMSO 0.1% (one‐way ANOVA for (A) and (C), Mann–Whitney for (B) and (D))

CCl_4_ was used as a NASH disease model, inducing a fibrotic phenotype responsible for alterations in the blood vessel architecture. PCLS obtained from CCl_4_‐treated animals showed a remarked reduction in the amount of serotonin‐induced contraction. CCl_4_‐treated animals only had 5% contractility, while 16% contraction was seen in PCLS from healthy animals (Figure 6A–D)). Pre‐incubation with 10 µM Ketanserin was able to block the serotonin‐induced contraction (Figure 6B). Riociguat or BI 703704, prevented serotonin‐induced contraction in PCLS of healthy animals (Figure 6A and B). Riociguat and BI 703704 were also able to prevent the 5% vessel contraction induced by serotonin in PCLS from CCl_4_ animals (Figure 6C and D).

### 3.5 Only the sGC activator induces blood vessel relaxation in PCLS obtained from CCl_4_ animals

**FIGURE 7:**
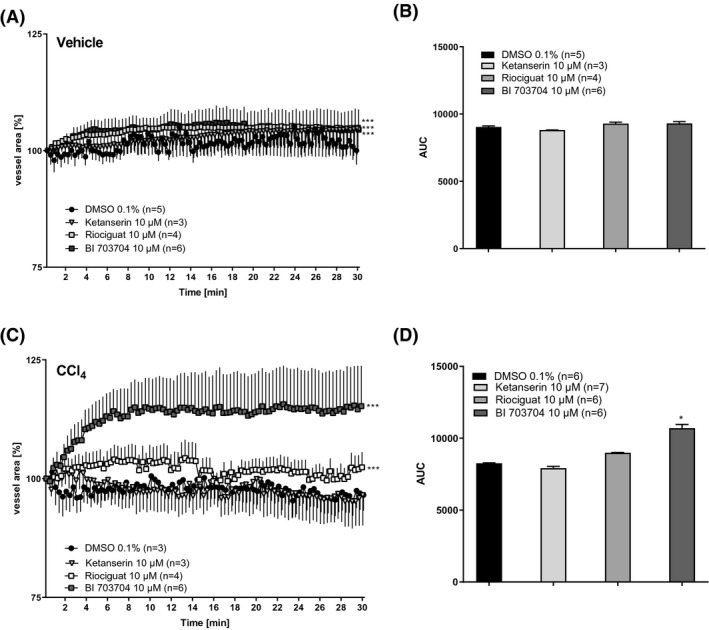
BI 703704 induces a more pronounced relaxation in CCl_4_‐treated animals compared to Riociguat. (A) Slices of vehicle‐treated animals were stimulated with 10 µM Ketanserin, Riociguat, or BI 703704 and blood area was followed for 30 min. Every 20 s a picture was taken and blood vessel area measured. (B) Area under the curve in vehicle‐treated animals (DMSO n:4, Ketanserin 10 µM n:2, Riociguat 10 µM n:4, BI 7030704 10 µM n:6). Technical loss of measurements has occurred. (C) Slices of CCl_4_‐treated animals were stimulated with 10 µM Ketanserin, Riociguat, or BI 703704 and blood area was followed for 30 min. Every 20 s a picture was taken and blood vessel area measured. (D) Area under the curve in CCl_4_‐treated animals (DMSO n:3, Ketanserin 10 µM n:3, Riociguat 10 µM n:4, BI 7030704 10 µM n:6). Technical loss of measurements has occurred. *p < .05, **p < .01, ***p < .001 compared to DMSO 0.1% (One Way ANOVA for (A) and (C), Mann–Whitney for (B) and (D))

To assess the relaxation potential of the sGC compounds under fibrotic conditions, PCLS from CCl_4_‐treated rats were analyzed. Relaxation was measured as an increase in blood vessel area compared to the area at the start of the incubation. Ketanserin was not able to relax blood vessels in PCLS from either vehicle or CCl_4_‐treated animals (Figure 7A and C). Riociguat and BI 703704 induced relaxation in blood vessels obtained from both vehicle and CCl_4_‐treated animals, however the most pronounced effect in the induction of relaxation by sGC compounds were seen in the CCl_4_ group (Figure 7B and D).

Riociguat showed a relaxation of 3% while BI 730740 is showing a relaxation of 15% compared to the DMSO control. The relaxation reached a maximal effect after 8 min and remained stable until the end of the experiment.

## 4 DISCUSSION AND CONCLUSION

In NASH patients the hepatic vascular resistance is increased due to structural changes in the liver related to fibrosis. An increased resistance as observed in fibrotic or cirrhotic patients is additionally due to an increase in contractile agents such as endothelin‐1, serotonin, and simultaneously a diminished availability of the relaxant agent NO.^6^, ^31^ Both endothelin‐1 and serotonin induce a contractile response and are known to regulate blood flow in both the portal and sinusoidal vessels and increase the portal pressure in rats.^11^ In cirrhotic patients, higher plasma levels of serotonin are observed and inhibition leads to a reduced portal pressure in these patients.^6^ These literature data provide evidence for the importance of serotonin in cirrhotic portal hypertension and that modulating the serotonin pathway might be beneficial in the treatment of portal hypertension.

**FIGURE 8:**
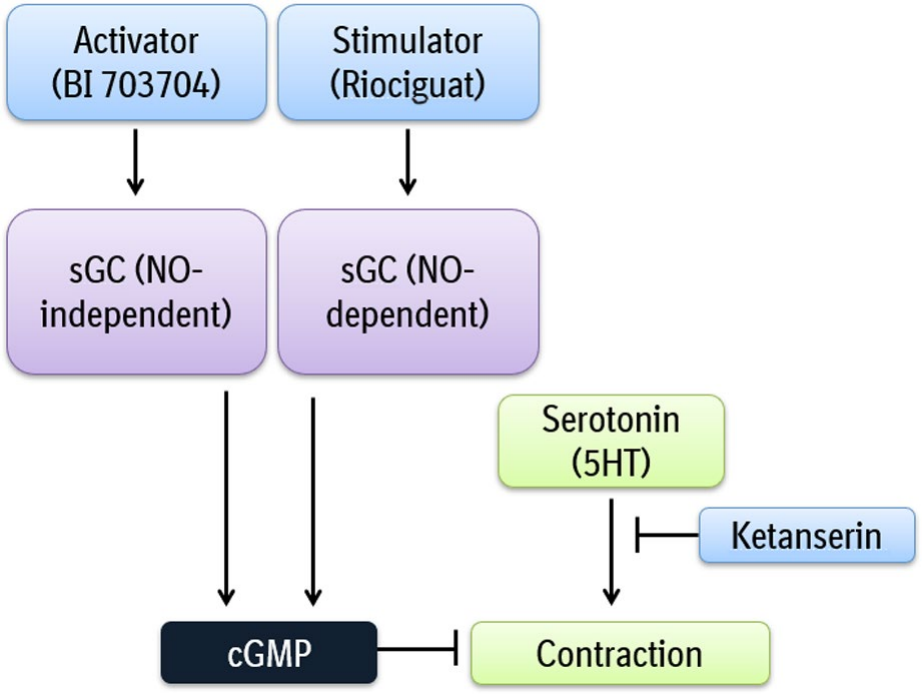
Overview of the effect of an sGC stimulator and sGC activator on blood vessel contraction

Our new ex vivo model based on PCLS (Figure 1) showed that Ketanserin, a vasodilator, prevents liver blood vessel contraction induced by serotonin (Figures 2 and 8) consistent with a prior report,^11^ thus qualifying our ex vivo model as a pre‐clinical method that allows for analysis of contractile and relaxant function of blood vessels in the liver.

Further, the enhanced contraction potential of endothelin downstream of the 5‐HT has been previous shown by Bhaskaran et al. where a synergistic effect on contraction was observed in rat aortic rings when ET‐1 was administered after serotonin stimulation.^32^

Analyzing the effect of a drug on vasoconstriction or vasorelaxation in PCLS may have the potential to model the changes in resistance and blood pressure within the blood vessel seen in vivo. In comparison to preclinical in vivo model, this ex vivo model based on PCLS is less time‐consuming and provides the potential to evaluate multiple experimental conditions in tissue from one animal.

Furthermore, the measure of portal pressure or hepatic venous pressure gradient (HVPG)^33^ as performed in in vivo experiments is invasive and low throughput. Instead, the ex vivo model described here provides a framework supporting the 3R principles as multiple mechanisms can be tested in tissue from one animal, reducing animal numbers and allowing the refinement of in vivo studies according to developed insights from the ex vivo model.

A limitation of the ex vivo model is the lack of focus on the portal vein. An identification of the veins is only possible based on staining performed after contraction or relaxation measurements necessitating that both central veins and portal veins are included in our ex vivo model. Unfortunately, closure of the vessel during contraction measurement makes it difficult to re‐analyze the vessel after staining. The effect of serotonin seems to be similar in the portal and central vein, so the amount of contraction cannot be used as an identification method for the different veins.^11^, ^34^ One way to optimize the identification is using a higher resolution microscope to differentiate between the different veins without staining. Other constraints concern the viability of the slice which can only be assessed after pre‐incubation therefore multiple slices are needed to allow for a sufficient group size at the end. Lastly, blood vessels in PCLS from severely diseased animals have the tendency to be closed, increasing the difficulty in performing contraction experiments.

Overall, this model based on PCLS is suited to test the effect of contractile agents, such as serotonin and ET‐1 (Figure 2), and to test the inhibitory properties of compounds, which makes the model suitable for analyzing therapeutic concepts, such as portal hypertension. The use of the model for drug discovery as shown here is based on the link between serotonin and the NO/sGC/cGMP pathway. We demonstrated that both sGC compounds can prevent serotonin‐induced contraction in PCLS from naïve rats (Figures 3 and 8). This result is consistent with the results obtained using Riociguat in in vivo models. In a bile duct ligation (BDL) study, Riociguat was able to reduce portal pressure in rats^35^ indicating that the ex vivo method described can be predictive for in vivo experiments (Figure 3). Based on the data presented by Brusilovskaya et al, we believe that a PDE‐inhibitor like Tadalafil, which inhibits portal pressure in a BDL rat model could also inhibit blood vessel contraction as seen with our PCLS method.^20^ However, we speculate that targeting the upstream components such as sGC might induce a greater effect than targeting the downstream inhibition of cGMP degradation by PDE inhibition. We hypothesized that the effect of the sGC activator will become more evident in the presence of oxidative stress. Therefore, CCl_4_ treatment in vivo was used to induce a fibrotic phenotype with associated oxidative stress response as shown by the expression profiling of the NRF2 pathway. Compared to the vehicle group, CCl_4_‐treated animals have an increased expression of NQO1, a cytoprotective gene, which is upregulated in the presence of ROS.^36^

The CCl_4_ treatment of rats also induced alterations in the expression of the different subunits of the sGC enzyme. The increase of both the α1 and β1 subunit in parallel to a decrease in the β2 subunit observed in the CCl_4_ model (Figure 5) might result in an increased activity of the α1/β1 form and increased activity upon sGC activation as seen under fibrotic conditions.^37^

In Figure 6 we demonstrate that the contractile effect of serotonin was almost absent in liver slices from CCl_4_‐treated animals (Figure 6A and C), which could point to an increased level of vasoconstriction due to liver injury.^38^ This is consistent with the findings of Ruddell and colleagues that the hepatic artery only shows a minor increase in serotonin‐induced contraction in cirrhotic patients compared to healthy patients.^11^ The plasma of patients with liver cirrhosis and portal hypertension showed an 18‐fold increase of serotonin making the blood vessel less sensitive to exogenously added serotonin.^11^

To analyze the effect of both the sGC activator and the sGC stimulator on relaxation, we used a different setup, in which PCLS were incubated with the compound directly and monitored by video‐microscopy. We were unable to induce relaxation with any of the compounds in PCLS of the vehicle‐treated rats (Figure 7A and B), potential due to maximal relaxation in these vessels. However, the sGC activator BI 703704, but not the sGC stimulator Riociguat, was able to induce relaxation in PCLS of CCl_4_‐treated animals (Figure 7C and D). This supports the hypothesis of an sGC activator having beneficial effects under conditions of oxidative stress compared to an sGC stimulator.

In conclusion, the ex vivo method based on PCLS described here captures aspects of the NASH phenotype as seen in humans and can be used to study blood vessel contractility in the liver, evaluate potential therapeutic agents as shown for the sGC pathway (Figure 8) and allow for translation of therapeutically findings to pre‐clinical animals models.

## 5 DISCLSOURE

The authors declare that they have no known competing financial interests or personal relationships that could have appeared to influence the work reported in this paper.

## 6 ETHICS APPROVAL

The animal studies were conducted according to approved animal licenses of Boehringer‐Ingelheim and conform to national guidelines for animal welfare.

## AUTHOR CONTRIBUTIONS

Anouk Oldenburger: Conceptualization, Methodology, Writing ‐ Original Draft. Gerald Birk: Methodology, Software. Marco Schlepütz: Methodology. Andre Broermann: Methodology. Birgit Stierstorfer: Methodology, Investigation. Steven S. Pullen: Writing ‐ Review & Editing. Jörg F. Rippmann: Conceptualization. Writing ‐ Review & Editing, Supervision.

## Supporting information

Figure S1

## Data Availability

The authors confirm that the data supporting the findings of this study are available within the article [and/or] its supplementary materials. The data that support the findings of this study are available from the corresponding author upon reasonable request.
