# Supplementary figures and images for: Modulation of vascular contraction via soluble guanylate cyclase signaling in a novel ex vivo method using rat precision‐cut liver slices
Source: Pharmacol Res Perspect. 2021 May 20;9(3):e00768. doi: 10.1002/prp2.768 (PMC8135082; doi:10.1002/prp2.768)

**a**

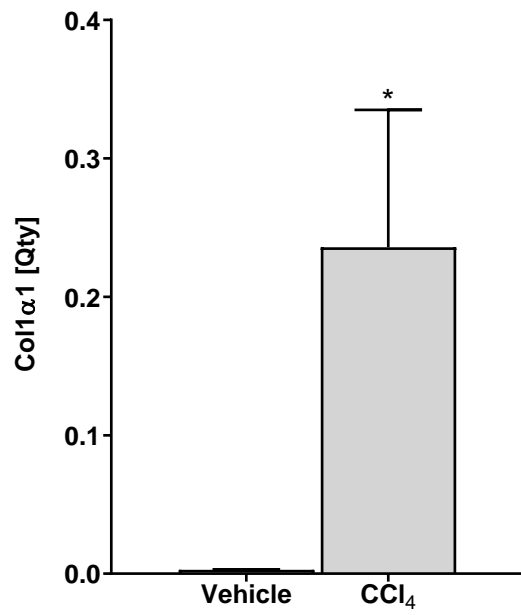

**b**

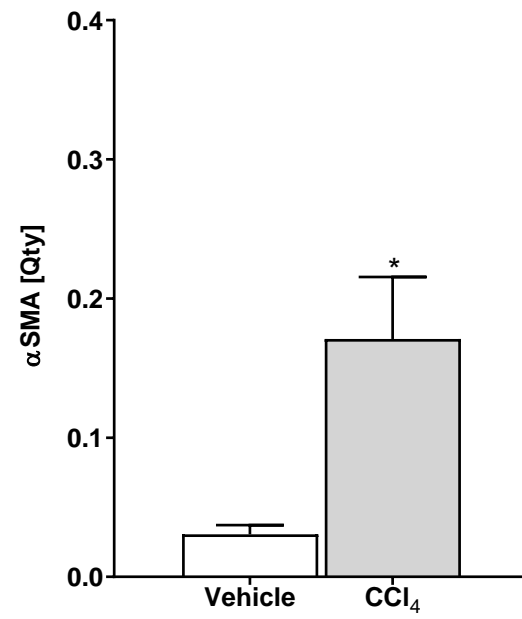

Supplement: Supplementary file 1 — Figure S1 [file PRP2-9-e00768-s001.pdf]
